# Effects of CFTR-ENaC on spinal cord edema after spinal cord injury

**DOI:** 10.1515/med-2024-1082

**Published:** 2024-11-12

**Authors:** Guowei Shen, Yunpeng Zhang, Xinkun Cheng, Dongdong Li, Zhiyong Ding, Jiwei Tian, Hui Chen, Huiming Ding

**Affiliations:** Department of Orthopaedics, BenQ Medical Center, The Affiliated BenQ Hospital of Nanjing Medical University, Nanjing, 210019, Jiangsu, China; Department of Orthopaedics, BenQ Medical Center, The Affiliated BenQ Hospital of Nanjing Medical University, No. 71, Hexi Street, Jianye District, Nanjing, 210019, Jiangsu, China

**Keywords:** LPS, CFTR-ENaC, spinal cord edema, spinal cord injury, PI3K/AKT

## Abstract

**Objective:**

To explore the role of cystic fibrosis transmembrane conduction regulator (CFTR)-Epithelial sodium channel (ENaC) in spinal cord edema after spinal cord injury (SCI) and the related mechanism.

**Methods:**

Lipopolysaccharide (LPS)-treated M1830 astrocytes were applied as the SCI *in vitro* model. Immunohistochemistry, real-time PCR, and Western blotting were utilized to detect CFTR and ENaC expression. Enzyme-linked immunosorbent assay was used to measure inflammatory cytokines including TNF-α, IL-1β, IL-6, and IL-18. Transmission electron microscope examined ultrastructure changes, while CFTR-172 or Capsazepine treatment assessed their effects on edema and inflammation. Western blot analysis was employed to evaluate the PI3K, p-PI3K, AKT, and p-AKT signaling pathways in treated cells.

**Results:**

LPS-treated M1830 cells exhibited increased levels of CFTR and pro-inflammatory cytokines, including TNF-α, IL-1β, IL-6, and IL-18, alongside decreased ENaC expression and suppressed p-PI3K/PI3K and p-AKT/AKT levels. Degeneration of the myelin sheath and axons was observed in LPS-treated M1830, while changes in ultrastructural were recovered after adding CFTR-172 or Capsazepine. The level of CFTR, TNF-α, IL-1β, IL-6, and IL-18 was decreased, while the level of ENaC, p-PI3K/PI3K, and p-AKT/AKT was increased obviously in LPS-treated M1830 with CFTR-172, Capsazepine, or IGF-1.

**Conclusion:**

Down-regulation of CFTR and up-regulation of ENaC can attenuate inflammation in SCI by activating the PI3K/AKT signaling pathway, highlighting a new therapeutic approach for SCI treatment. These findings address a critical gap in current SCI treatments and suggest a novel intervention strategy targeting ion channel regulation.

## 1 Introduction

Spinal cord injury (SCI) means the injury to any part of the spinal cord, leading to changes in the body functions below the injured part [1], affecting more than 2.5 million people worldwide [2]. SCI comprises primary injury and secondary injury [3]. Primary injury involves irreversible damage to the spinal cord caused by the cutting, compression, and impact on the spinal cord with the spinal fracture or dislocation. Secondary injury refers to a series of pathophysiological changes occurring based on primary injury, including hemorrhage, edema, accumulation of inflammatory factors, and excitatory toxins. SCI is usually a progressive process. Secondary injury further aggravates local spinal cord tissue compression, thrombosis, and ischemia, leading to progressive neuronal death [4], causing serious consequences. The World Health Organization reports that SCI patients face elevated risks of depression and mortality, imposing substantial economic burdens on families and society [5]. Therefore, understanding the mechanisms underlying SCI and developing effective treatment strategies is crucial for improving patient outcomes.

Spinal cord edema is a typical feature of the pathological progression of SCI and represents a critical therapeutic target [6]. A previous study reported that spinal cord edema occurred in the acute stage of SCI and lasted for several weeks [7]. The time and degree of formation and regression of spinal cord edema are closely related to the repair and prognosis of SCI. The accumulation of blood and water in the spinal cord after injury will increase the intrathecal pressure and the disturbance of intramedullary microcirculation, leading to aggravated tissue damage and loss of motor function [8]. Current clinical management of spinal cord edema relies mainly on conservative treatment approaches, which often fail to adequately prevent edema progression. Thus, there is an urgent need to explore novel therapeutic targets and develop interventions to control spinal cord edema after SCI.

Edema in SCI is classified into vasogenic and cytotoxic edema [9]. Vasogenic edema results from increased permeability of the blood–brain barrier (BBB) due to disrupted tight junctions and due to disrupted tight junctions by fluid extravasation into the spinal cord parenchyma. Cytotoxic edema, driven by astrocyte dysfunction and ion imbalance, involves intracellular accumulation due to impaired sodium-potassium pump activity [10]. This cytotoxic form plays a pivotal role in secondary SCI, serving as a precursor to vasogenic edema [11]. Astrocytes, the main type of neuroglial cells, play a key role in the progress and metabolism of the central nervous system (CNS), regulating cell volume and ion homeostasis between cells, and maintaining the BBB. Astrocyte swelling is an important feature of spinal cord cytotoxic edema, accompanied by IL-1β, and the formation of various cytokines including IL-6 and HMGB1 [12] is related to the functional recovery and prognosis after SCI. However, the molecular mechanism and regulatory factors of astrocyte swelling after SCI are still unclear. Further study on the mechanism of astrocyte swelling is helpful to better reveal the cytopathological changes of SCI.

The mechanism of edema formation in SCI is related to the imbalance of CNS water metabolism. The water metabolism balance of the human normal CNS is determined by osmotic pressure and hydrostatic pressure. Ion channels and aquaporin play an irreplaceable role in the water metabolism balance of CNS. In recent years, research on the mechanism of spinal cord edema has focused on the AQP family. AQPs can play a role in spinal cord edema by influencing CNS water metabolism and transportation and facilitating astrocyte migration. However, the research results show that the role of AQPs in the pathogenesis of SCI is biphasic and controversial, which cannot fully explain the mechanism of spinal cord edema, and there are still difficulties in treatment, so it is urgent to find new molecular targets. As an important molecule for regulating water balance, the mechanism of ion channels in spinal cord edema after SCI is slightly lacking. Ion channels are expressed in the nervous system, and distributed in choroid plexus epithelial cells, periventricular ependymal epithelial cells, and astrocytes. They are the main cellular basis for water transport and regulation between glial cells, cerebrospinal fluid, and blood vessels, and play an important role in maintaining the balance of water metabolism in the CNS. Research shows that under the influence of hypoxia, ischemia, poisoning, and other factors, the function of the cell membrane ion channel is disordered, and the concentration of ions and osmotic pressure in cells are increased, which promotes the liquid to enter cells, causing cytotoxic edema [13]. The imbalance of CNS water metabolism is crucial to spinal cord edema development. Ion channels and aquaporins play key roles in maintaining this balance but have not been thoroughly explored in the context of SCI [13]. Research shows that inhibition of sodium potassium chloride cotransporter 1 and AQP4 at the same time can better prevent spinal cord edema and destruction of spinal cord tissue after SCI [14]. While the above studies have focused on aquaporins, their role in SCI remains controversial and inadequately explains the mechanisms of edema formation.

Ion channels, including Cystic fibrosis transmembrane conduction regulator (CFTR) and Epithelial sodium channel (ENaC), are integral to regulating CNS water transport and have been implicated in various forms of edema. CFTR, which is expressed on epithelial cells’ apical capsule, is an anion channel activated by cAMP, mediates Cl^−^ transport in epithelial cells, and maintains water metabolism balance and body cavity secretion. CFTR is the most widely studied in the respiratory system and is considered to play a significant role in the occurrence and progress of pulmonary diseases such as pulmonary edema by mediating alveolar fluid clearance. Lack of functional CFTR can lead to chronic pulmonary inflammation [15]. In animal experiments, it was observed that after chronic lipopolysaccharide (LPS) exposure, CFTR-deficient mice had enhanced pulmonary inflammatory response accompanied by increased cytokine expression [16]. The role of CFTR in the edema of target organs has also been observed in other systems: in the mouse model of chlamydia trachomatis infecting the uterus, the upregulation of CFTR expression will activate the inflammatory factor NF-κB-mediated IL-1β It leads to the accumulation of uterine cavity fluid and the aggravation of edema, which can be reversed by chlamydia trachomatis-specific antibiotics and CFTR blockers [17]. In ovarian tissue, excessive injection of gonadotropin caused an abnormal increase in serum estradiol level in rats, significantly increased the expression of CFTR in the ovary, caused ovarian interstitial edema, multiple follicular cysts and luteal cysts, and induced ovarian hyperstimulation [18]. At present, the study of CFTR in the nervous system is still in its infancy. The localization study shows that CFTR is expressed in a variety of peripheral and CNS and is widely expressed at a relatively constant level in human spinal cord and sympathetic ganglion cells [19]. However, there is little research on the relevant mechanism. Whether CFTR plays a role in spinal cord edema after SCI is still unclear, which is worth further study.

ENaC is another important ion channel protein that regulates water metabolism. It is responsible for the rate-limiting reabsorption of Na^+^ and plays an important role in maintaining the balance of Na^+^, extracellular fluid volume, and blood pressure stability. Respiratory research shows that in LPS-induced lung injury, insulin can up-regulate ENaC through PI3K/SGK1 pathway, reduce pulmonary edema, and protect pulmonary epithelium [20]. High estrogen down-regulates the expression of ENaC and affects the fluid transport of endometrial epithelial cells, which partly explains the phenomenon of low embryo implantation rate in a high estrogen environment [21]. In the mouse model with abnormal water and sodium metabolism in the kidney, it was confirmed that hyperlipidemia can cause water and sodium retention by down-regulating the expression of ENaC in the kidney through SGK1 [22].

As two important ion channel proteins, CFTR and ENaC regulate each other. It was first observed that Na^+^ transport disorder was associated with abnormal expression of ENaC in cystic fibrosis patients. It is speculated that the functional effect of ENaC depends on the activation of CFTR. The fluid secretion mediated by CFTR and the fluid absorption balance mediated by ENaC play a key role in maintaining the airway surface fluid stability, and ENaC is considered to be a treatment target to improve the mucus transport of respiratory epithelial cells in CF patients [23]. CFTR and ENaC are involved in the balance of intestinal epithelial secretion and absorption, and the dysfunction of these important transporters explains the pathogenesis of various forms of diarrhea [24]. In addition, in the male reproductive system, it was observed that CFTR and ENaC were both expressed in the testes and vas deferens and were regulated by testosterone. They were involved in maintaining the stability of the male reproductive tract to maintain normal male fertility [25]. However, the role and mechanism of CFTR and ENaC in spinal cord edema after SCI, as well as the interaction between them, are unclear. The further study of CFTR and ENaC in the occurrence and progress of spinal cord edema is of major significance to improve the prognosis of spinal cord edema and find new therapeutic targets.

Hence, our study proposed the hypothesis: after SCI, the expression of CFTR in spinal cord astrocytes was up-regulated and the expression of ENaC was down-regulated, leading to astrocyte swelling, affecting the balance of spinal cord water metabolism, thus inducing and aggravating the occurrence of spinal cord edema, resulting in numerous adverse consequences of SCI. This study aims to investigate the roles of CFTR and ENaC in SCI-induced spinal cord edema, providing a novel perspective on the molecular mechanisms underlying secondary injury.

## 2 Materials and methods

### 2.1 Reagents

Enzyme-linked immunosorbent assay (ELISA) kits for the detection of TNF-α, IL-1β, IL-6, and IL-18 were obtained from Beyotime (Beyotime, Beijing, China). Specific antibodies against CFTR, ENaC, PI3K, p-PI3K, AKT, p-AKT, and GAPDH were purchased from Cell Signaling Technology (Beverly, MA, USA). CFTR-172 (CAS No: 307510-92-5) and Capsazepine (CAS No: 138977-28-3) were obtained from Sigma (Sigma, Burlington, MA, USA). IGF-1 was obtained from Sino Biological Inc. (Beijing, China). LPS was obtained from Beyotime (Beyotime, Beijing, China).


**Human and animal rights**: No animals/humans were used for studies that are the basis of this research.

### 2.2 Cell culture and treatments

Mouse Astrocytes-spinal cord (M1830) was purchased from Shanghai Zhong Qiao Xin Zhou Biotechnology Co., Ltd. The cells were characterized by immunofluorescence with antibodies specific to GFAP and tested free of mycoplasma, bacteria, yeast, and fungi. M1830 cells were cultured in astrocyte medium in a coated dish under 5% CO_2_ at 37°C. M1830 cells were treated with 100 ng/mL-LPS, 100 ng/mL-LPS + CFTR-172 (20 μmol/L), 100 ng/mL-LPS + Capsazepine (5 μmol/L), or 100 ng/mL-LPS + IGF-1 (1 µg/10 µL) for 24 h.

### 2.3 Immunohistochemical examination

To examine the CFTR and ENaC expression in M1830 cells, immunohistochemistry (IHC) was conducted. Briefly, paraffin-embedded M1830 cell slides were treated with hydrogen peroxide (3%) with methanol and then blocked by 10% goat serum. The slides were washed three times and incubated primary antibodies (CFTR, 1:2,000; ENaC, 1:2,000) overnight and then HRP-labeled IgG (1:500, ab6721) (Abcam, MA, USA). After that, the slides were stained with DAB and observed under a microscope (IX73, Olympus, Tokyo, Japan).

### 2.4 Transmission electron microscopy (TEM)

TEM was applied to observe exosomes. Briefly, M1830 cells were resuspended and applied to copper grids for 10–30 s. Coverslips were mounted with drops of 2% phosphotungstic acid, pH 7.0, on the same slide for 5 s. After 20 min, the grid was dry and was observed by using a TEM (Hitachi, Tokyo, Japan).

### 2.5 Quantitative reverse transcription PCR (qRT-PCR)

TRIzol reagent (T9424, Sigma-Aldrich, Beijing, China) was applied to extract RNA from cells. cDNA synthesis was performed using a Reverse Transcription Kit (RR037Q, Takara, Dalian, China). The PCR system (25 μL) comprises 0.3 μM primers for each, 12.5 μL SYBR Green quantitative polymerase chain reaction (qPCR) Master Mix (A46012, Invitrogen, ThermoFisher Scientific), and 2.5 μL cDNA. Primers were as follows: CFTR (mouse):5′-CTGGACCACACCAATTTTGAGG-3′ and 5′-GCGTGGATAAGCTGGGGAT-3′, ENaC (mouse): 5′-TGTGTCCAGCTACAAACCAATG-3′ and 5′-CAT CATGCCCACTTCGTAACA-3′, GAPDH (mouse): F-5′-GGTTGAGCAGGTACTTT-3′ and R-5′-AGCAAGTGCACAAGAGGAAG-3′. qPCR analysis was conducted on a MyiQ2 PCR thermocycle instrument (Sigma-Aldrich, Beijing, China). The expression level of GAPDH was used to standardize mRNA expression and use multiple variation = 2^−ΔΔCT^ calculation.

### 2.6 Western blot

Cells were dissociated by using RIPA buffer. The concentration of total proteins was detected by using a commercialized kit (Beyotime, Beijing, China). Total proteins (60 μg) were separated by 15% sodium dodecyl sulfate polyacrylamide gel electrophoresis and then transferred to a PVDF membrane (ISEQ00010, Sigma-Aldrich, Beijing, China). Milk powder (5%) diluted with Tris buffer brine containing Tween 20 (0.05%) was used to block the nonspecific sites (37573, TBST, Thermo Scientific, Beijing, China). Then, the PVDF membranes were incubated by using the primary antibodies overnight at 4°C: mouse polyclonal antibodies for CFTR (1:2,000 dilution), ENaC (1:1,000 dilution), PI3K (1:1,000 dilution), p-PI3K (1:2,000 dilution), AKT (1:2,000 dilution), p-AKT (1:2,000 dilution), and GAPDH (1:1,000 dilution). After the PVDF membranes were washed three times with TBST, they were incubated with HRP-conjugate goat anti-mouse secondary antibody (A0208, 1:1,000 dilution; Beyotime, Beijing, China). Blots were evaluated by enhanced chemiluminescence. GAPDH was applied as an internal reference. The level of the above protein levels was quantified densitometrically using Quantity One software (Bio-Rad, Hercules, USA).

### 2.7 Measurement of TNF-α, IL-1β, IL-6, and IL-18 by ELISA

The concentrations of TNF-α, IL-1β, IL-6, and IL-18 in cells were analyzed using commercialized ELISA kits (Beyotime Biotechnology, Beijing, China). A microplate reader (BMG LABTECH, Offenburg, Germany) was applied to determine the absorbance.

### 2.8 Statistical analysis

SPSS (IBM SPSS Statistics 19.0) was used for all statistical analyses. Data were presented as mean ± SD. *t*-Test was applied to analyze the differences between the two groups. One-way analysis of variance was used to analyze the differences among three or more groups. *P* < 0.05 indicated that the difference is considered statistically significant.

## 3 Results

### 3.1 The expression of CFTR and ENaC in LPS-treated M1830 cells

**Figure 1:**
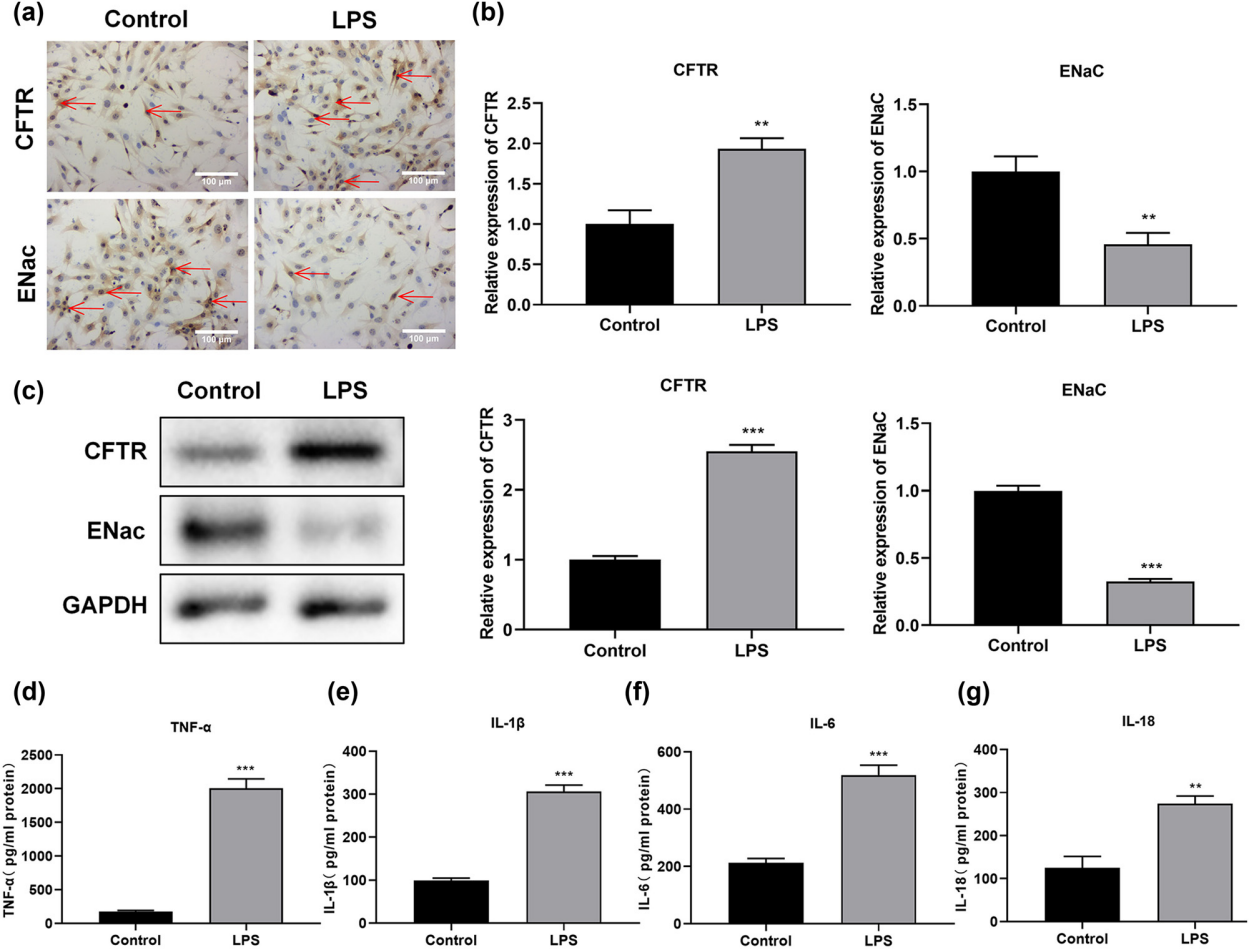
The expression of CFTR and ENaC and the concentration of TNF-α, IL-1β, IL-6, and IL-18 in LPS-treated M1830 cells. (a) The expression of CFTR and ENaC by IHC. Scale bar = 100 μm. (b) The expression of CFTR and ENaC by real-time PCR. (c) The expression of CFTR and ENaC by Western blot. (d) The level of TNF-α by ELISA. (e) The level of IL-1β by ELISA. (f) The level of IL-6 by ELISA. (g) The level of IL-18 by ELISA. *N* = 3. **P < 0.01, ***P < 0.001.

CFTR expression was increased in LPS-treated M1830 cells, while ENaC expression was down-regulated in LPS-treated M1830 cells by IHC (Figure 1a). CFTR expression was increased significantly in LPS-treated M1830 cells, however, the level of ENaC was down-regulated significantly by real-time PCR (*P* < 0.01) and Western blot (*P* < 0.001) (Figure 1b and c).

### 3.2 The level of TNF-α, IL-1β, IL-6, and IL-18 in LPS-treated M1830 cells

The level of TNF-α (*P* < 0.001), IL-1β (*P* < 0.001), IL-6 (*P* < 0.001), and IL-18 (*P* < 0.01) was increased significantly in LPS-treated M1830 cells by ELISA (Figure 1d–g).

### 3.3 The ultrastructural changes in LPS-treated M1830 cells after adding CFTR-172 or Capsazepine

**Figure 2:**
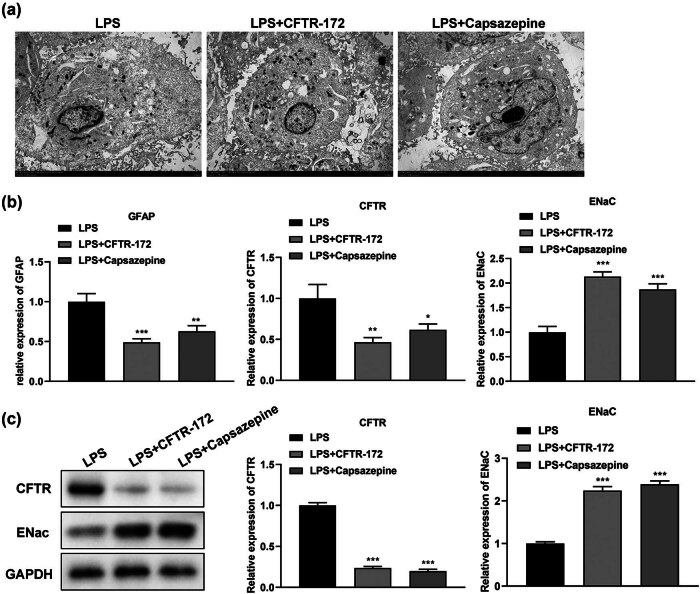
The microstructural alterations and expression of CFTR and ENaC in LPS-treated M1830 cells treated with CFTR-172 and Capsazepine. (a) Microstructural analysis in M1830 cells by TEM. (b) The expression of GFAP, CFTR, and ENaC by real-time PCR. (c) The expression of CFTR and ENaC by Western blot. *N* = 3. *P < 0.05, **P < 0.01, ***P < 0.001.

Compared with the LPS-treated group, the degree of cell swelling was decreased after adding CFTR-172 or Capsazepine (Figure 2a).

### 3.4 The expression of CFTR and ENaC in LPS-treated M1830 cells after adding CFTR-172 or Capsazepine

The level of CFTR was down-regulated significantly in LPS-treated M1830 cells with CFTR-172 or Capsazepine; however, the expression of ENaC was increased significantly by real-time PCR (*P* < 0.05) and Western blot (*P* < 0.001) (Figure 2b and c).

### 3.5 The level of TNF-α, IL-1β, IL-6, and IL-18 in LPS-treated M1830 cells after adding CFTR-172 or Capsazepine

**Figure 3:**
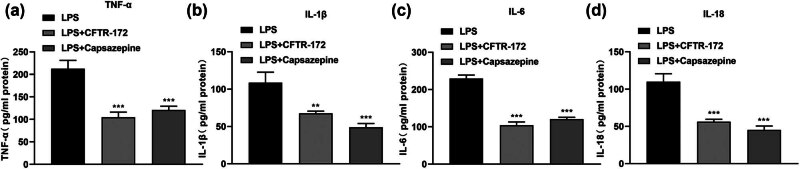
The concentration of TNF-α, IL-1β, IL-6, and IL-18 in LPS-treated M1830 cells treated with CFTR-172 and Capsazepine. (a) The level of TNF-α by ELISA. (b) The level of IL-1β by ELISA. (c) The level of IL-6 by ELISA. (d) The level of IL-18 by ELISA. *N* = 3. **P < 0.01, ***P < 0.001.

The level of TNF-α (*P* < 0.001), IL-1β (*P* < 0.01), IL-6 (*P* < 0.001), and IL-18 (*P* < 0.01) was decreased significantly in LPS-treated M1830 cells with CFTR-172 or Capsazepine by ELISA (Figure 3a–d).

### 3.6 The expression of PI3K, p-PI3K, AKT, and p-AKT in LPS-treated M1830 cells with or without IGF-1

**Figure 4:**
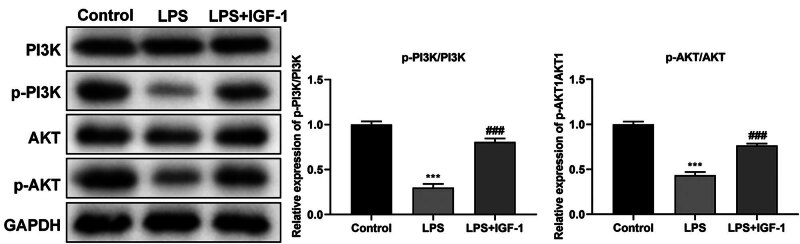
The expression of PI3K, p-PI3K, AKT, and p-AKT in LPS-treated M1830 cells with or without IGF-1 by Western blot. *N* = 3. ***P < 0.001. ^###^ *P* < 0.001 (LPS + IGF-1 vs LPS).

The ratio between the level of p-PI3K and PI3K was decreased significantly in LPS-treated M1830 cells (*P* < 0.001), while the ratio was recovered after adding IGF-1 (*P* < 0.001). The ratio between the level of p-AKT and AKT was decreased significantly in LPS-treated M1830 cells (*P* < 0.001), while the ratio was recovered after adding IGF-1 (*P* < 0.001) (Figure 4).

### 3.7 The expression of PI3K, p-PI3K, AKT, and p-AKT in LPS-treated M1830 cells with or without CFTR-172, Capsazepine, or IGF-1

**Figure 5:**
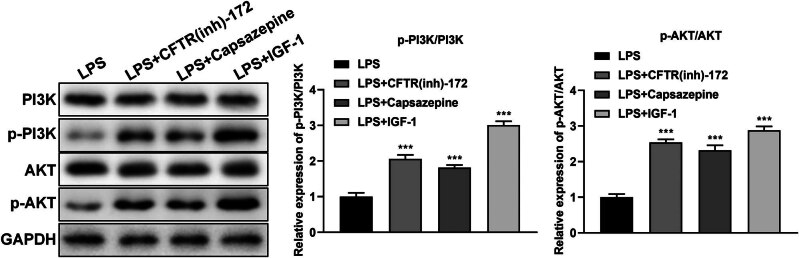
The expression of PI3K, p-PI3K, AKT, and p-AKT in LPS-treated M1830 cells with or without CFTR-172, Capsazepine or IGF-1 by Western blot. *N* = 3. ***P < 0.001.

Compared with the LPS group, the ratio between the level of p-PI3K and PI3K was increased significantly in LPS-treated M1830 cells with CFTR-172, Capsazepine, or IGF-1 (*P* < 0.001). The ratio between the level of p-AKT and AKT was also increased significantly in LPS-treated M1830 cells with CFTR-172, Capsazepine, or IGF-1 (*P* < 0.001) (Figure 5).

## 4 Discussion

SCI refers to the structural and functional damage of the spinal cord caused by various pathogenic factors, leading to the loss of neurological function [26,27]. Due to the need for long-term wheelchair use and long-term complications, it has been a heavy burden on the patient’s physiology and psychology [28]. The pathogenesis and treatment of SCI have always been the focus of clinical attention [29]. Spinal cord edema is the main pathological progress of SCI and the duration and degree of spinal edema directly affect the repair and prognosis of SCI [30]. Therefore, it is important to inhibit the occurrence and development of spinal cord edema timely and effective for the treatment of SCI. Besides, we need to understand the mechanism of spinal cord edema.

A previous study has shown that CFTR is involved in regulating anion transport and mucociliary foreign body clearance in the airway. Deactivation of CFTR function leads to mucus retention and chronic infection in the airway, followed by inflammation in the local airway, causing lung damage [31]. Although, there was no direct evidence to clarify the relationship between CFTR and SCI. LPS stimulation in alveolar macrophages increased CFTR expression [32]. In our study, the expression of CFTR was increased in LPS-treated M1830 cells (Figure 1a–c), which showed consistent results with the findings in the above study. Increased CFTR was related to the occurrence and development of edema [33]. We also found edema in LPS-treated M1830 cells by TEM (Figure 2a). Proinflammatory cytokines including TNF-α, IL-1β, IL-6, and IL-18 have been reported to participate in the process of SCI [34]. Here, the level of TNF-α, IL-1β, IL-6, and IL-18 was increased in LPS-treated M1830 cells (Figure 1d–g), which showed consistent results with the findings of the previous study [35]. After adding CFTR-172, the expression of CFTR (Figure 2b–c) and the level of TNF-α, IL-1β, IL-6, and IL-18 (Figure 3a–d) were recovered, which confirmed that CFTR was involved in the process of spinal cord edema in SCI.

ENaC is an ion channel protein that regulates water metabolism. It reduces the rate of Na^+^ reabsorption and plays a significant role in maintaining Na^+^ balance and extracellular fluid volume [36]. ENaC has been confirmed to be related to the development of SCI [37]. A previous study has shown that SCI decreased the activity of ENaC in the animal model. In our study, we also found the expression of ENaC was decreased in LPS-treated M1830 cells (Figure 1a–c), which showed consistency with the above results [38]. After adding Capsazepine, the expression of ENaC (Figure 2b and c) and the level of TNF-α, IL-1β, IL-6, and IL-18 (Figure 3a–d) were recovered, which confirmed that ENaC was also involved in the process of spinal cord edema in SCI. It is worth mentioning that Capsazepine has an antagonistic effect on the TRPV1 channel, which also helps to alleviate pain and inflammation [39]. This article mainly focused on the effect of Capsazepine on ENac and did not study TRP channels. As a part of the changes after SCI, TRP channels have important effects on inflammation and pain relief. We do not have a comprehensive understanding of the complex interplay among SCI, ENac, and TRPV1. Future studies could explain this system by incorporating a more in-depth consideration of TRPV1, as well as by using a more comprehensive experimental design.

PI3K/AKT signal pathway is involved in the pathological process of SCI. The activation of this pathway can reduce the inflammatory reaction and the permeability of the blood-spinal cord barrier, prevent neuronal apoptosis, and promote the recovery of neural function [40,41]. In the present study, we found that the PI3K/AKT signaling pathway was inhibited in LPS-treated M1830 cells, while it was recovered after adding IGF-1 (Figure 4a), indicating that the PI3K/AKT signaling pathway was involved in the process of SCI. Compared with the LPS-treated group, the PI3K/AKT signaling pathway was activated after adding CFTR-172, Capsazepine or IGF-1 (Figure 5a), which indicated that CFTR-172, Capsazepine, and IGF-1 could promote the SCI recovery. However, this study was conducted using an *in vitro* model, which may limit the generalizability of the findings. To fully understand the effects of CFTR-ENaC on spinal cord edema following SCI, it is essential to establish an animal mode in future research. This approach would facilitate a more comprehensive assessment of the physiological relevance and potential therapeutic implications of our findings.

## 5 Conclusion

In conclusion, this study is the first to demonstrate that the down-regulation of CFTR and up-regulation of ENaC can decrease inflammation associated with SCI by activating the PI3K/AKT signaling pathway. These findings could provide new insights into potential therapeutic strategies for managing SCI. However, several limitations exist, including the need for an animal model to validate the effects of CFTR and ENaC on spinal cord edema. Future studies should explore the interactions between ENaC, CFTR, and TRPV1, utilizing a more comprehensive experimental design to fully elucidate these mechanisms.

## Abbreviations

LPS: lipopolysaccharide
SCI: spinal cord injury
IHC: immunohistochemistry
TEM: transmission electron microscope
WHO: World Health Organization
BBB: blood–brain barrier
CNS: central nervous system
NKCC1: sodium potassium chloride cotransporter 1
CFTR: cystic fibrosis transmembrane conduction regulator
ENaC: epithelial sodium channel
